# Influence of Transglutaminase Crosslinking on Casein Protein Fractionation during Low Temperature Microfiltration

**DOI:** 10.3390/foods10123146

**Published:** 2021-12-18

**Authors:** Ritika Puri, Francesca Bot, Upendra Singh, James A. O’Mahony

**Affiliations:** 1 School of Food and Nutritional Sciences, University College Cork, T12 TP07 Cork, Ireland; francesca.bot@ucc.ie (F.B.); sa.omahony@ucc.ie (J.A.O.); 2 Lakeland Dairies, Bailieborough, A82 N6K8 Co. Cavan, Ireland; singhu@lakeland.ie

**Keywords:** microfiltration, low temperature, crosslinking, transglutaminase, β-casein, enzyme

## Abstract

Low temperature microfiltration (MF) is applied in dairy processing to achieve higher protein and microbiological quality ingredients and to support ingredient innovation; however, low temperature reduces hydrophobic interactions between casein proteins and increases the solubility of colloidal calcium phosphate, promoting reversible dissociation of micellar β-casein into the serum phase, and thus into permeate, during MF. Crosslinking of casein proteins using transglutaminase was studied as an approach to reduce the permeation of casein monomers, which typically results in reduced yield of protein in the retentate fraction. Two treatments (a) 5 °C/24 h (TA) and (b) 40 °C/90 min (TB), were applied to the feed before filtration at 5 °C, with a 0.1 µm membrane. Flux was high for TA treatment possibly due to the stabilising effect of transglutaminase on casein micelles. It is likely that formation of isopeptide bonds within and on the surface of micelles results in the micelles being less readily available for protein-protein and protein–membrane interactions, resulting in less resistance to membrane pores and flow passage, thereby conferring higher permeate flux. The results also showed that permeation of casein monomers into the permeate was significantly reduced after both enzymatic treatments as compared to control feed due to the reduced molecular mobility of soluble casein, mainly β-casein, caused by transglutaminase crosslinking.

## 1. Introduction

Microfiltration (MF) can be applied to achieve a wide range of operations in the dairy industry, including bacteria removal [1], fat removal and milk fat globule membrane separation. Fractionation of casein and whey proteins in skim milk is regarded as one of the applications with the greatest potential for this technology [2]. Traditionally, MF is performed at processing temperatures between 50–55 °C, which offers low viscosity and relatively high flux [3,4]. In addition, high temperature prevents solubilisation of micellar casein, principally β-casein, into the serum phase by way of strong hydrophobic interactions within micelles, and thereby limiting the permeation of casein into permeate [5]. However, despite these advantages, the use of high temperatures brings significant challenges during industrial MF, which can include undesirable growth of thermophilic microorganisms in the filtration system leading to negative impacts on plant hygiene, pH reduction and filtration performance [6]. Performing MF at low temperature (5–15 °C) is considered as an alternative approach to addressing these processing challenges and has gained momentum in recent years. Low temperature processing ensures significantly reduced microbial growth [6], and enzyme activity [7,8], thereby providing higher quality process streams and products [9]. Moreover, during low temperature processing MF plant require a lower number of cleaning cycles with associated benefits in terms of consumption of water and cleaning agents, significantly reducing the environmental impact of such processes [6]. At low temperature, the decrease in hydrophobic protein interactions in milk and increase in solubility of colloidal calcium phosphate promotes the reversible dissociation of micelle bound casein, principally β-casein, into the serum phase in monomeric form [10,11,12]. These monomeric forms of casein are smaller in size and therefore can more readily permeate through the MF membranes (typically 0.1 µm pore size) [13].

Some of the applications of low temperature MF include isolation of β-casein [14,15] and development of β-casein-enriched native whey for nutritional applications, owing to the ability of such proteins to achieve more human milk-like protein profile [16]. Apart from these applications, the majority of MF operations aim to optimise casein retention during milk protein fractionation to achieve greater yield by reducing losses of casein in the permeate, and developing highly pure whey protein ingredients from the corresponding permeate streams [17]. However, it is challenging to restrict serum casein permeation at low temperature by simply altering processing conditions such as transmembrane pressure (TMP) and concentration factor [18]. Recently, Schiffer et al. [17] studied the effects of calcium chloride addition to skim milk on release of casein monomers into the permeate, during MF at 10–20 °C. It was found that 5–10 mM calcium chloride was required to achieve considerable reduction in release of casein monomers from micellar to serum phase, and thereby into permeate. Further addition of calcium chloride to skim milk resulted in deposit formation on the membrane, which negatively impacted the ratio of casein to whey proteins in permeate.

A possible alternative approach to study the temperature dependent impact on micellar casein, and thus on partitioning of caseins during MF at low temperature could be to use enzymatic crosslinking by microbial transglutaminase (mTG). mTG is a crosslinking enzyme capable of forming inter- and intra-molecular crosslinks between and within proteins, respectively [19,20]. Casein proteins are excellent substrates for mTG action, primarily due to their flexible nature with little or no secondary structure, in contrast to globular whey proteins [21]. The mTG crosslinking of casein leads to the formation of higher molecular weight protein molecules such as dimers, trimers, oligomers and polymers [22]. These molecular changes contribute to improved techno-functional properties in yogurt [23,24], cheese [25] and milk powders [26]. The studies relating to mTG crosslinking of micellar casein concentrate and the subsequent effects on its functional properties are limited; however, milk protein concentrate (MPC) powder crosslinked with mTG (0.5 g/L, 30 °C, 24 h) has been found to have improved wettability and water sorption [27]. Moreover, viscosity increase was also shown to be controlled in MPC suspension (10% *w/w*) with added calcium chelating salts after crosslinking with mTG [28]. The activity of mTG on individual caseins is influenced by the accessibility of individual caseins within micellar systems [29]. The κ-casein, and to some extent β-casein, are predominant at the micellar surface, which makes them more susceptible to mTG, while the α_s_-caseins are largely embedded within the micellar structure [29]. However, when relevant environmental conditions of casein micelles are modified, for example, by altering the temperature, the susceptibility of each casein to mTG activity is modified. Hinz et al. [20] has shown that on incubating reconstituted skim milk with mTG at 5, 20 and 40 °C, β-casein showed the highest susceptibility towards mTG at 5 °C followed by 20 and 40 °C. The susceptibility of α_s_-casein was reported to be independent of incubation temperature, while the susceptibility of κ-casein increased as a function of incubation temperature.

Nonetheless, the ability of β-casein to undergo temperature dependent reversible dissociation from casein micelles and its susceptibility towards mTG crosslinking has not been studied previously in the context of milk protein fractionation at low temperature, as a technological solution to reduce loss of casein during MF of skim milk. Therefore, in this study reconstituted micellar casein concentrate feed rich in casein micelles was enzymatically crosslinked with mTG under two different environmental conditions, (a) while cooling at 5 °C for 24 h (TA) and (b) while warming at 40 °C for 90 min (TB). The time–temperature combinations selected were based on previous literature relating to susceptibility of casein proteins to crosslinking [20,30,31]. In particular, temperature considerably affects the physicochemical nature of casein micelles in terms of hydrophobic interactions and calcium phosphate solubility [12], which in turn can influence the susceptibility of individual caseins to mTG crosslinking. The mTG demonstrates optimum enzyme activity at temperature ranging between 40 and 50 °C, at pH 6.0 [32] and can induce a high degree of casein crosslinking in a short period of time, depending on the enzyme concentration [33]. Therefore, to achieve extensive crosslinking in a short period of time, 40 °C/90 min was selected [33]. For TA treatment, 5 °C was selected because it represents a temperature at which partial solubilization of casein can take place [13] when milk is cooled. The higher incubation time (24 h) for TA treatment was selected to counteract the effect of low temperature on enzyme activity and to achieve similar extent of crosslinking as TB treatment [20,30,31]. The industrial MF of skim milk is typically performed at either high processing temperature ~45–55 °C or low temperature ~10–15 °C [3,4,6,17], therefore two incubation temperatures, 5 and 40 °C, which are of practical relevance to industrial dairy processing were shortlisted. To study the influence of mTG crosslinking of protein on fractionation and filtration performance, the crosslinked feed samples were subjected to MF at 5 °C.

## 2. Materials and Methods

### 2.1. Materials

Micellar casein concentrate (MCC) (Milei^®^ MC 88) powder was kindly provided by MILEI GmbH, Leutkirch, Germany. Microbial transglutaminase, Activa^®^ MP (EC 2.3.2.13), was kindly provided by Ajinomoto Foods Europe SAS (Paris, France). All standards were purchased from Sigma-Aldrich (Arklow, Wicklow, Ireland). All chemicals and reagents were of analytical grade.

### 2.2. Sample Preparation and mTG Crosslinking

Three independent feed batches, of volume 6 L, were prepared for three filtration experiments. MCC powder was initially reconstituted at 30 g/L in demineralized water at 40 °C, using a high-speed mixer at 8000 rpm for 60 min, after which 0.5 g/L of sodium azide was added to the feed to prevent microbial growth. After high-speed mixing, reconstituted feed was stirred at 350 rpm at 45 °C for 3.5 h. Before placing the feed at 4 °C, stirring for 48 h at 400 rpm, to ensure complete hydration of the casein micelles, 2 mM of calcium chloride was added [34], to provide an ionic environment similar to that of milk serum. The fully rehydrated feed was then sub-divided into 3 separate batches of 2 L each. One batch was treated as control (CTR), while the other two were subjected to enzymatic treatment.

For both treatments, the same amount of enzyme powder (7.5 g powder/L feed batch) was reconstituted into the feed to achieve ~25.0 units of mTG/g protein. The higher enzyme concentration (25.0 units of mTG/g protein) was selected to induce a greater degree of crosslinking [22,33] and to investigate predominantly its effect on filtration and protein fractionation. The mTG enzyme powder had declared activity of 100 units/g powder. For treatment (a) TA, feed was incubated at 5 °C for 24 h and for treatment (b) TB, feed was incubated at 40 °C for 90 min. The TA, TB and CTR feed were immediately heat treated at 80 °C for 5 min by placing in a water bath with gentle stirring to inactivate the enzyme and subsequently cooled to 20 °C by placing in an ice bath, followed by overnight storage at 4 °C until filtration on the following day. Samples were collected from all three feed batches for further analysis before starting filtration experiments.

### 2.3. Microfiltration of Feed

Microfiltration (MF) experiments were performed as described by Puri et al. [8]. A pressure-driven bench scale crossflow filtration rig and Durapore MF membrane cassette with nominal pore size of 0.1 µm, enclosed within a stainless-steel membrane holder (Pellicon 2 mini-holder) was procured from Merck-Millipore (Tullagreen, Cork, Ireland). Before using the membrane, it was initially cleaned as per the instructions provided by the membrane manufacturer. After initial cleaning, normalized water permeability (NWP) of clean water was measured for a new membrane at transmembrane pressure (TMP) of 0.1 bar at 25 °C. The initial NWP of 900 L/m^2^/h/bar was compared as a benchmark for NWP measurements taken before and after membrane cleaning during each MF run. To understand the influence of mTG crosslinking of protein on permeate flux, the filtration was carried out in full recirculation mode by returning the retentate and permeate lines back to the feed vessel. The feed flow was kept constant during filtration in all experiments. The feed temperature was maintained throughout filtration at 5 ± 0.5 °C, by recirculating retentate back into the feed vessel. The retentate line passed through a plate heat exchanger that received water in alternate channels supplied by a water bath set at 3.5 °C. After initial feed flow equilibration of 15 min to achieve the steady flow and target feed temperature of 5 °C, the TMP was set at 0.2 bar for 90 min filtration cycle. After 5 min of feed equilibration at set TMP, permeate flow was measured volumetrically, in duplicate, by recording the volume of permeate at intervals of 5 min, and flux was measured. The filtration performance was assessed in terms of flux and casein concentration in the permeate. After filtration, the MF membrane and filtration rig were flushed thoroughly with de-ionised water, and standard cleaning protocol was followed as recommended by the membrane manufacturer.

### 2.4. Analysis

#### 2.4.1. Proximate Analysis and Color

CTR, TA and TB feeds were analysed for total nitrogen using the Kjeldahl method with a nitrogen-to-protein conversion factor of 6.38 [35]. Ash content was determined by dry ashing in a muffle furnace at 500 °C for 5 h [36]. Total solids were measured by oven drying at 103 °C for 5 h. The pH of the feed samples was measured immediately before MF filtration and for the permeate samples just after 90 min filtration cycle using a pH meter (bench pH meter, model HI2211-02, Hanna Instruments Ltd., Bedfordshire, UK) at 25 ± 1 °C. The colour of feed samples (15 mL) was measured using a chromameter CR-400 (Konica Minolta Sensing, Inc, Osaka, Japan) using CIELAB coordinates (L*, a*, b*). The feed samples were maintained at 5 °C before duplicate analysis. The chromameter was calibrated before the measurement using a white tile. In the CIELAB system, L* value corresponds to the brightness and the values can vary between 0 (black) and 100 (white), a* value measures degree of redness (positive values) or greenness (negative values), and b* value measures degree of yellowness (positive values) or blueness (negative values).

#### 2.4.2. Apparent Viscosity

The apparent viscosity of feed and permeate samples (20 mL) was measured at 100/s for 60 s, within 2 h of the filtration runs, at 5 °C, using a HAAKE RotoVisco 1 viscometer (Thermo Scientific, Dieselstrasse 4, D-76227, Karlsruhe, Germany) equipped with a cup and bob geometry [8]. The samples were analysed in duplicate from three independent runs.

#### 2.4.3. Turbidity

The fresh permeate samples from CTR and mTG treated feeds were analysed for turbidity development over 5 to 55 °C within 1 h of the filtration run. The absorbance was measured at a wavelength of 600 nm using a Cary 100 Bio UV–visible Spectrophotometer (Varian Inc., Palo Alto, CA, USA) [37]. Samples were incubated for 30 min each at 5, 25, 40 and 55 °C, before reading the absorbance value in the spectrophotometer. The spectrophotometer was equipped with a temperature control system which was adjusted accordingly to achieve the incubation temperature prior to the absorbance measurement. All samples were measured in duplicate at each incubation temperature for three independent filtration runs.

#### 2.4.4. SDS-Polyacrylamide Gel Electrophoresis

Protein profile of feed and permeate samples was qualitatively assessed using sodium dodecyl sulphate–polyacrylamide gel electrophoresis (SDS-PAGE) with precast gels (Mini-PROTEAN TGX, Bio-Rad Laboratories, Irvine, CA, USA) under reducing conditions using an AcquaTank mini gel unit (Acquascience, Bellbrook Industrial Estate, Uckfield, UK) [8]. The final protein concentration of feed for loading was 1 mg/mL and permeate was used as it came; feed and permeate volumes of 5 and 15 µL were loaded onto the gels. Along with samples, 5 µL of each α_s_-casein, β-casein and κ-casein protein standard and low heat skim milk powder of 1 mg/mL concentration were also loaded. All gels were Coomassie-stained and were scanned using a desktop scanner (HP Scanjet G4010, HP, Leixlip, Ireland).

#### 2.4.5. Reversed-Phase High Performance Liquid Chromatography

The protein profile of the feed and permeate samples were measured using RP-HPLC (Agilent 1220 Infinity II LC, Santa Clara, CA 95051, USA) with a C18 column (3.6 μm × 250 mm × 4.6 mm, Aeris Widepore, Phenomenex, UK), using solvents A (10.0% acetonitrile, 89.9% ultrapure water and 0.10% TFA) and B (89.9% acetonitrile, 10.0% ultrapure water and 0.10% TFA); the injected sample volume was 40 μL, and detection was performed at 214 nm. The sample was mixed with buffer at a 1:1 ratio, followed by filtration through 0.45 μm filters (Minisart^®^ RC25, G¨ottingen, Sartorius AG, Germany). The concentrations of individual proteins were determined by preparing standard curves (R^2^ > 0.99) of the respective proteins and results were expressed as mg protein/mL of sample.

### 2.5. Statistical Analysis

The data is reported as average ± standard deviation of at least duplicate analysis of samples from three independent filtration runs. The raw mean values were statistically analysed using one-way analysis of variance (ANOVA). Means were tested for statistical significance (*p* < 0.05) by applying Duncan’s post hoc test, using the software IBM SPSS Statistics 20.0 (IBM Corp. Released 2011. IBM SPSS Statistics for Windows, Version 20.0. Armonk, NY, USA).

## 3. Results and Discussion

### 3.1. Extent of Casein Crosslinking with mTG in Micellar Casein Concentrate Feed

The total nitrogen content of CTR, TA and TB feed were comparable (*p* > 0.05) in the range of 2.9–3.0 g/kg. The total solids content of TA and TB feed were significantly higher (*p* < 0.05) than CTR, due to the added enzyme powder. The ash content was comparable (*p* > 0.05) in all feeds ranging between 0.28–0.29 g/kg. The feed pH was measured at the start of MF runs and was significantly (*p* < 0.05) higher for TA and TB, than CTR feed. The action of mTG includes three reactions, crosslinking between glutamine and lysine amino acids, amine incorporation and deamidation [21]. These reactions release free ammonia as a byproduct which can increase pH [21]. No significant difference was observed in apparent viscosity of feeds, with values ranging between 3.1–3.4 mPa.s. It is consistent with the findings of Mounsey et al. [33], who reported that control and mTG treated (10 g/kg, 5 min, 40 °C) micellar casein solutions (25 g protein/kg) at pH 6.7 showed Newtonian flow behaviour with comparable values of 3.06 and 3.11 mPa.s, for apparent viscosity, measured at a shear rate of 100 s^−1^ and 22 °C. The incubation of micellar casein with mTG at neutral pH is reported to induce intramolecular crosslinking, while intermolecular crosslinking which predominantly occurs at micellar surface and contributes to viscosity development is reported to be limited at neutral pH [33]. Table 1 shows colour values for CTR and treated feeds; the L* value, which is a measure of whiteness, ranged between 80.3–82.2, with a significantly (*p* < 0.05) lower value for TA than CTR and TB feed. The negative a* and b* values, representative of green and blue, respectively, differ significantly (*p* < 0.05) among all three feeds, with lowest a* and b* values recorded for TA. The colour differences observed between the feed samples, especially TA and TB feeds, regardless of the same feed origin and same amount of added enzyme, suggest that these colour differences may have resulted from mTG induced changes in the feed.

**Figure 1:**
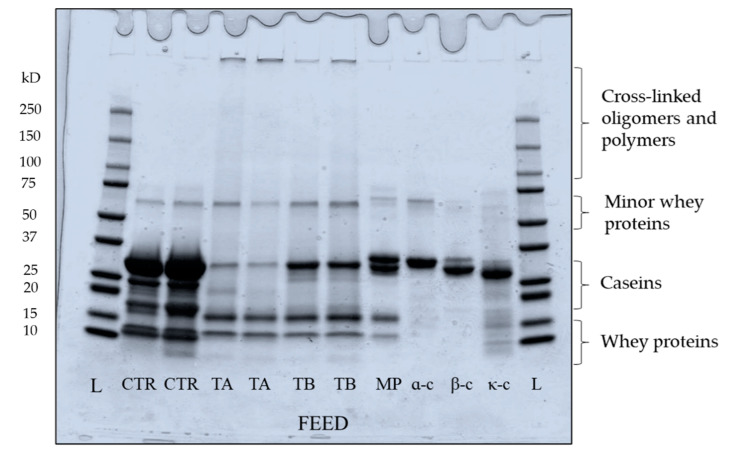
Electrophoretic patterns under reducing conditions of mTG crosslinked micellar casein concentrate feeds used in microfiltration at 5 ± 0.5 °C. Bands represent samples from two independent runs at each treatment. L: ladder; CTR: Control; TA: 5 °C/24 h; TB: 40 °C/90 min; MP: low heat skim milk powder; α-c: α_s_-casein; β-c: β-casein; κ-c: κ-casein.

Figure 1, which represents the electrophoretic pattern of feed samples, shows marked changes in casein proteins in both TA and TB feeds mediated by enzymatic crosslinking. In addition, in TA and TB the mTG crosslinking caused the formation of higher molecular weight dimers, trimers, oligomers and polymers [19], which can be seen located at higher molecular weight regions in both TA and TB feeds. Although the distinct effect of mTG was seen in both TA and TB feed, some proteins were not crosslinked, simply because of unavailability of reactive lysine and glutamine residues for mTG action [29], owing to the hydrophobic/electrostatic interactions and nanoclusters of colloidal calcium phosphate within casein micelles [11]. In TA feed, β- and κ-casein bands are largely not visible, suggesting their extensive crosslinking. At low temperature, casein micelles demonstrate swelling and become more open due to low hydrophobic interactions which can facilitate extensive crosslinking within the micelles, as seen in TA feed. The higher susceptibility of β-casein towards mTG at low temperature (TA feed) can be linked to its greater flexibility within the micelle, due to its ability to reversibly dissociate at low temperature [11,38,39]. This is in accordance with the results of Duerasch et al. [29] who also reported high reactivity of micellar β-casein. Although κ-casein is the least susceptible substrate for mTG due to its lower number of lysine and glutamine residues [21,40], it is also extensively crosslinked in TA feed. It is due to its readily accessible location on the micellar surface, as it is reported that extent of crosslinking is highly influenced by structural conformation of casein micelles [20]. In TB feed, low intensity bands of β-casein and κ-casein are visible, suggesting that some caseins were not crosslinked. At high temperature (40 °C), β-casein remains associated with the micellar phase and strong hydrophobic/electrostatic interactions take place within micelles, limiting the availability of lysine and glutamine residues for mTG crosslinking. The α_s_-caseins, which are primarily located in the inner regions of micelles [41], are least susceptible to mTG action, which can also support the finding reported in Figure 1, showing the presence of α_s_-casein bands in both TA and TB feed. It suggests that susceptibility of α_s_-caseins to mTG is less temperature dependent than for the other two caseins [19]. The whey proteins in TA and TB feeds were largely unaffected by mTG action (Figure 1) with limited extent of crosslinking [21]. The feed was prepared from cold microfiltered MCC powder, which mostly consisted of native and globular whey proteins, with a casein to whey protein ratio of 96:4. The globular whey proteins are poor substrates for mTG in their native form, while they are more prone to crosslinking after heat-induced denaturation or high pH-induced unfolding [32].

**Figure 2:**
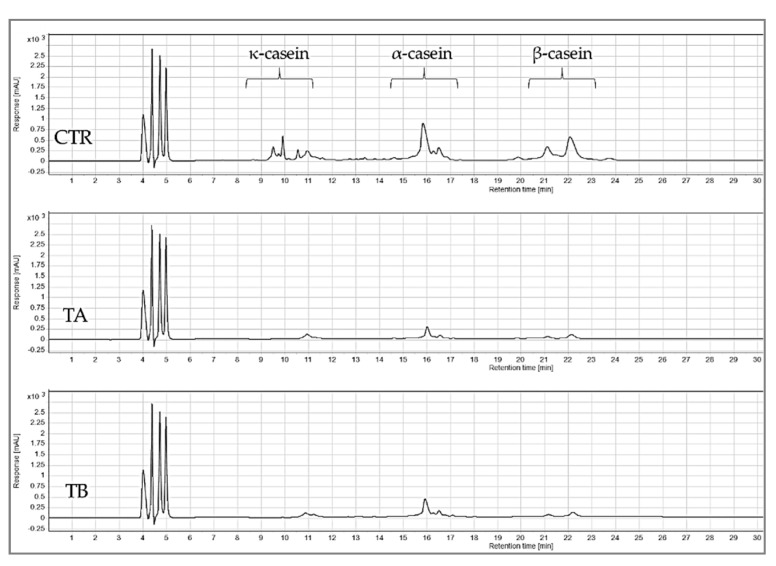
Chromatograms from reversed-phase high performance liquid chromatography analysis of mTG crosslinked micellar casein concentrate feed used in microfiltration. CTR: no enzyme treatment; TA: 5 °C/24 h; TB: 40 °C/90 min.

**Figure 3:**
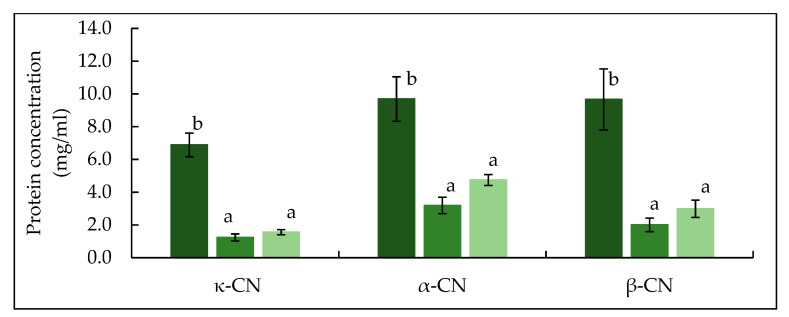
Concentration of κ-casein, α_s_-casein and β-casein in mTG crosslinked micellar casein concentrate feed used in microfiltration. CTR: no enzyme treatment (■); TA: 5 °C/24 h (■); TB: 40 °C/90 min (■). Different lower-case letters indicate significant differences (*p* < 0.05) between proteins.

The RP-HPLC chromatograms in Figure 2 shows the peaks identified for κ-, α_s_- and β-casein, while no peaks were visible for whey proteins due to their concentration being below the detection limit. [33]. Based on the quantification of identified peaks (Figure 3), both TA and TB feeds had significantly (*p* < 0.05) lower concentrations of κ-, α_s_- and β-casein compared to CTR feed. Between the two treatments no significant (*p* > 0.05) difference was observed in the concentrations of κ-, α_s_- or β-casein.

### 3.2. Influence of Feed Crosslinking on Microfiltration Performance

**Figure 4:**
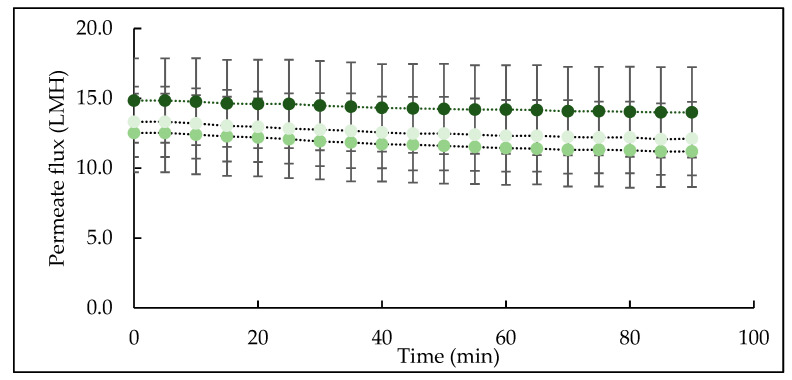
Permeate flux as a function of processing time during microfiltration of micellar casein concentrate feed using a 0.1 µm membrane at 5 ± 0.5 °C. Line colour represents: CTR (•), TA (•), TB (•); Values are the means ± standard deviations of data from triplicate runs at each treatment. LMH: L/m^2^/h; CTR: no enzyme treatment; TA: 5 °C/24 h; TB: 40 °C/90 min.

The change in permeate flux as a function of filtration time is shown in Figure 4. The permeate flux declined during the first few minutes due to initial deposition [42], which then progressed and declined steadily throughout filtration for all three feeds. This is consistent with the findings of Hartinger and Kulozik [18], who also reported steady-state flux decline during cold MF of skim milk. The initial flux was in the range of 12−16 L/m^2^/h for all feed samples, which is higher than permeate flux of skim milk, 6 L/m^2^/h, at 4 °C, reported by France et al. [42]. The higher flux in the present study is mainly due to the nature of the starting feed material which was reconstituted micellar casein concentrate with low content of serum proteins which contribute significantly towards resistance to permeate flow during MF of skim milk [43]. Although no significant differences were observed among flux values in CTR, TA and TB feeds, TA feed had consistently high flux throughout filtration, followed by TB and CTR feeds. Since the processing conditions (temperature, TMP, crossflow velocity, dry matter and filtration duration) were kept constant for all feeds, the difference in flux can be linked to feed and its interactions with the MF membrane.

The intra- and intermolecular crosslinking has been reported to preserve the integrity of the micellar casein structure due to the formation of iso-peptide covalent bonds within the inner regions of the micelle (crosslinking of α_s_- and β-casein) [29]. The intra- and intermolecular crosslinking make the micelles largely insensitive towards technological treatments (e.g., high hydrostatic pressure) and destabilising agents (e.g., ethanol, urea and alkali) [44]. Moreover, formation of iso-peptide bonds in external regions and on the surface of the micelles (crosslinking of κ- and β-casein), also helps in preserving the micellar structure [45]. The higher flux of TA feed suggests that interactions between proteins themselves and with the membrane surface were limited (non-availability of reactive side chains due to crosslinking of micellar casein). This may have resulted in reduced surface adsorption and deposit formation which decreases the permeate flow resistance through the membrane during filtration. In TB feed, some proteins remained uncrosslinked (Figure 1) and may have contributed to greater resistance to permeation flow than TA feed.

**Figure 5:**
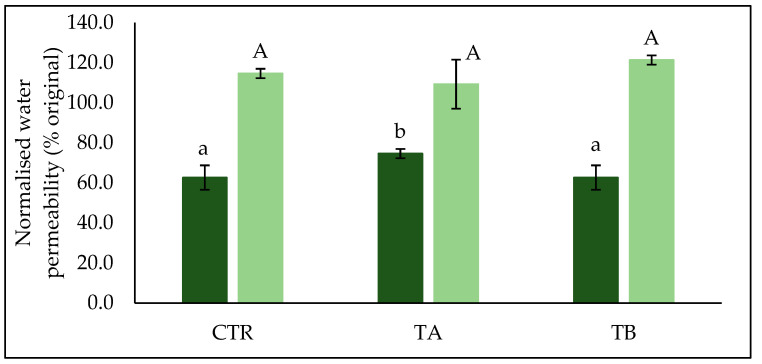
Normalised water permeability (% original value) of membrane measured before (■) and after (■) membrane cleaning process. Values are the means ± standard deviations of analysis from triplicate runs at each treatment. Different lowercase and uppercase letters on the bar graphs indicate significant differences (*p* < 0.05).

The extent of surface deposition and fouling was assessed by measuring water permeability of the membrane before and after cleaning (Figure 5). The NWP measured before cleaning (i.e., immediately after a filtration run) was significantly (*p* < 0.05) lower for TB (63%) and CTR (63%) feed, than for TA feed, which retained 75% of original permeability. The significantly (*p* < 0.05) higher NWP of TA feed corroborates the flux results and suggests that protein deposition and membrane fouling during filtration of TA feed was less intense than of TB and CTR feed. The significantly (*p* < 0.05) higher NWP for TA feed, suggests that β-casein, which was extensively crosslinked in TA feed, contributed to a lesser extent to protein adsorption on the membrane surface.

The NWP was measured again after cleaning and was shown to be restored, reaching up to 110–120% of the original value for all three feed samples (*p* > 0.05). During MF of skim milk at 4 °C, higher deposition of loosely bound fouling material can take place, but it can be effectively removed by hot water flushing [42,46]. In addition, the relatively low TMP applied throughout filtration (0.2 bar) can restrict the chances of irreversible deposition and fouling. Therefore, it implies that the cleaning regime followed was appropriate to remove deposits and foulants from the MF membrane and restore its NWP to continue its use.

### 3.3. Characteristics of Permeate Produced from Crosslinked Feed

#### 3.3.1. Composition of Permeate

The composition of permeate produced from CTR and treated feeds is shown in Table 2. The total solids of TA and TB permeate were comparable (*p* > 0.05), and significantly (*p* < 0.05) higher than CTR permeate. The total nitrogen was significantly (*p* < 0.05) lower in both TA and TB permeate, when compared to CTR. The ash content was low and not significantly (*p* > 0.05) different among the three permeates. The pH of TA and TB was comparable (*p* > 0.05), while significantly (*p* < 0.05) higher than for CTR permeate, in agreement with the high pH of TA and TB feeds (Table 1). The CTR permeate had significantly higher viscosity (2.4 ± 0.4 mPas) than TA permeate (1.8 ± 0.3 mPas), which might be due to its significantly (*p* < 0.05) higher protein content as represented by total nitrogen. The lowest viscosity of TA permeate can be linked to temperature-dependent crosslinking action of mTG on proteins in TA feed, particularly β-casein. The higher protein concentration in solution results in greater interactions which reduce space between molecules and causes lower water mobility and higher viscosity [47]. Moreover, β-casein is reported to have a high intrinsic viscosity at low temperature (5 °C) [48], which might explain the high viscosity of CTR permeate.

#### 3.3.2. Turbidity of Permeate Produced from Crosslinked Feed

**Figure 6:**
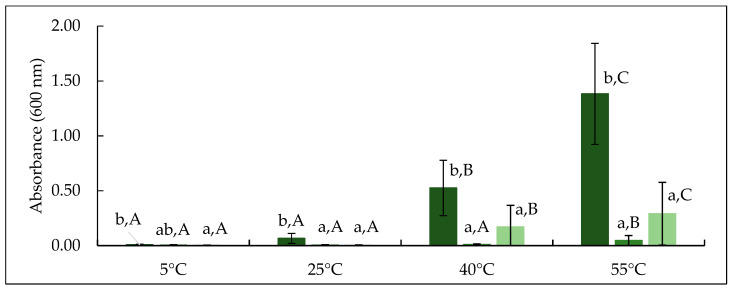
Absorbance at 600 nm of CTR, TA and TB permeate samples incubated at 5, 25, 40 and 55 °C. CTR: no enzyme treatment (■); TA: 5 °C/24 h (■); TB: 40 °C/90 min (■). Different lowercase letters indicate significant differences (*p* < 0.05) between treatment. Different uppercase letters represent significant differences (*p* < 0.05) between incubation temperature.

**Figure 7:**
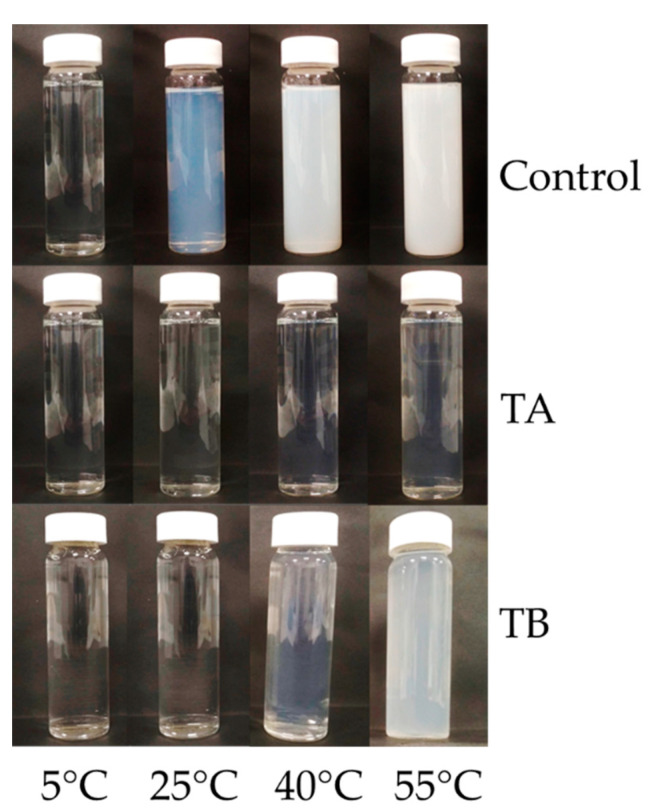
Visual appearance of turbidity development in CTR, TA and TB permeate samples heated at 5, 25, 40 and 55 °C. CTR: no enzyme treatment; TA: 5 °C/24 h; TB: 40 °C/90 min.

The development of turbidity in permeate was studied as an indication of β-casein concentration and its linked temperature dependent aggregation behaviour as influenced by mTG treatment. The absorbance of permeate produced from CTR, TA and TB feed was measured at 5, 25, 40 and 55 °C, as shown in Figure 6 and visual appearance of turbidity development is revealed in Figure 7. The absorbance of CTR increased significantly (*p* < 0.05) on increasing temperature from 25 to 55 °C. For TA and TB permeates, no significant (*p* > 0.05) increase in turbidity was observed up to 40 °C, while it increased significantly (*p* < 0.05) thereafter. On comparing CTR and treatments, at any given temperature, CTR permeate had significantly (*p* < 0.05) higher absorbance than TA and TB, while it was comparable (*p* > 0.05) between TA and TB. β-casein has a distinct temperature-dependent transition between micellar and monomeric states [11]; at low temperature (5−10 °C), β-casein generally exists in its monomeric form with size of approximately 8 nm [41]. With increasing temperature (>25 °C), β-casein can form micelle type structures with a hydrophobic core and a soft permeable exterior, with diameter up to 1 µm at 50 °C [39], resulting in high turbidity, as also observed in CTR permeate (Figure 7). In TA permeate, the turbidity remained visibly very low, suggesting that the proportion of monomeric β-casein was very low. On the other hand, when TB permeate was heated to 40 or 55 °C, the turbidity development was observed, which indicates the presence of monomeric β-casein that had ability to self-associate into aggregates on heating.

The temperature dependent behaviour of β-casein is strongly influenced by mTG crosslinking and is dependent on incubation conditions, as also reported by O’Connell and De Kruif [39]. O’Connell and De Kruif [39], studied crosslinking of isolated β-casein solution at low temperature (0 °C) and reported that after crosslinking, β-casein monomers lose their ability to self-micellise when they are heated to temperature >20 °C. This has been attributed to the decreased availability of lysine residues in the monomeric chains (due to mTG crosslinking) which modifies secondary structure of β-casein and reduces its mobility, due to the intramolecular crosslinking [49]. The secondary structure and molecular flexibility are essential for β-casein to reassociate with casein micellar phase or self-associate into micelles [39]. During TA treatment, the mTG crosslinking might have influenced the secondary structure and molecular mobility of soluble β-casein [29]. This mTG reaction can result in intramolecular crosslinking within mobile β-casein, which would either reduce its ability to reassociate with the micelle or restrict its self-micellisation [39]. The mTG reaction can also induce intermolecular crosslinking between monomeric β-casein and casein micelles via strong covalent iso-peptide bonds, which can limit the β-casein permeation through the membrane during low temperature MF [39].

At higher temperatures (40 °C) the hydrophobic interactions increase within casein micelles and most of the soluble casein (β-casein monomers) shifts towards the micellar phase, and it would be less available in serum phase for mTG action [12,41]. Therefore, crosslinking could only take place within micellar casein and it would be limited to proteins which have glutamine and lysine residues readily available for enzymatic reaction [40]. Due to hydrophobic and electrostatic interactions within casein micelles, some micellar protein (e.g., β-casein) would not crosslink (TB lane, Figure 1) [29]. Therefore, it may retain its ability to dissociate reversibly from the micellar phase to permeate through the membrane during low temperature MF as well as hold the ability to self-micellise on heating (Figure 7). This observation is not entirely in agreement with the findings of O’Connell and De Kruif [39], who reported that on crosslinking with mTG at 35 °C, β-casein micelles did not form monomers when temperature was lowered again; however, that study used pure β-casein, as opposed to casein micelles.

Overall, the results of the present study suggest that the comparable total nitrogen content of TA and TB permeate was principally contributed by β-casein, which was either crosslinked and lost its ability to form aggregates (TA permeate) or it was in monomeric form which could self-associate to form aggregates and contribute to turbidity development (TB permeate). These results may explain why there were visible differences between TA and TB permeates despite similar total nitrogen contents (Table 2).

#### 3.3.3. Protein Profile of Permeate Produced from Crosslinked Feed

**Figure 8:**
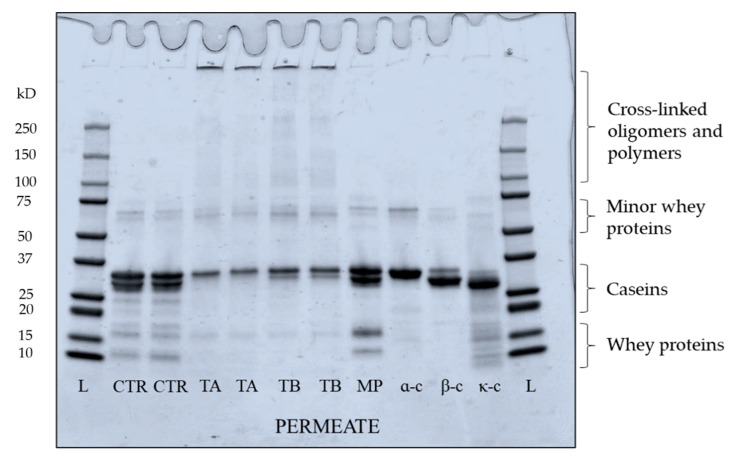
Electrophoretic pattern under reducing conditions of permeate samples produced by microfiltration of mTG crosslinked micellar casein concentrate feed at 5 ± 0.5 °C. Bands represent samples from two independent runs at each treatment. L: ladder; CR: CTR; TA: 5 °C/24 h; TB: 40 °C/90 min; MP: low heat skim milk powder; α-c: α_s_-casein; β-c: β-casein; κ-c: κ-casein.

**Figure 9:**
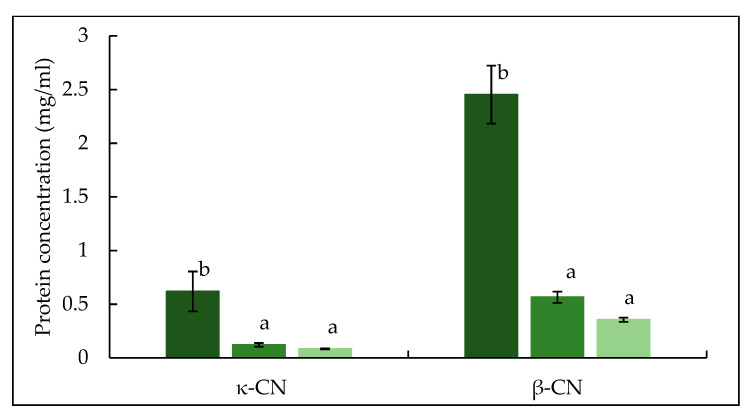
Concentration of κ-casein and β-casein in permeate samples produced during microfiltration of mTG crosslinked micellar casein concentrate feed at 5 ± 0.5 °C. CTR: no enzyme treatment (■); TA: 5 °C/24 h (■); TB: 40 °C/90 min (■). Different lowercase letters indicate significant differences (*p* < 0.05) between proteins.

The electrophoretic pattern of permeate produced from crosslinked feed is shown in Figure 8. Both TA and TB permeate contain bands for proteins which migrated as expected of native individual caseins, suggesting these proteins were not crosslinked by mTG. In TB permeate, a low intensity band of β-casein was visible, suggesting that some proportion β-casein (monomeric) was not crosslinked, supporting the finding from absorbance and turbidity development results. In both TA and TB permeate, κ-casein bands were not visible, while low intensity bands for α_s_-casein were identified. The low intensity bands of β-lactoglobulin and α-lactalbumin were also observed in TA and TB permeate (Figure 8), in agreement with the findings of Sharma et al. [50]. Sharma et al. [50] and Faergemand et al. [51], showed that both β-lactoglobulin and α-lactalbumin are susceptible to mTG action even without significant changes in their native structure. To quantify the concentration of individual protein in the permeate, RP-HPLC was used and peaks for β- and κ-casein were identified as shown in Figure 9. The concentrations of both β- and κ-casein were significantly (*p* < 0.05) higher in CTR permeate, while TA and TB permeate had comparable concentrations of β- and κ-caseins. No peaks for α_s_-casein were identified in any permeate sample, which is in agreement with the findings of France et al. [42], who studied the impact of low processing temperature (4 °C) on filtration performance and protein fractionation during MF of skim milk.

## 4. Conclusions

Crosslinking of casein micelles by mTG restricted the permeation of individual caseins, particularly β-casein, through a 0.1 µm membrane during cold MF of micellar casein concentrate. Although the crosslinking of casein micelles by mTG was temperature dependent, the impact on protein permeation was similar for both TA and TB treatments. The TA treatment demonstrated potential for reducing membrane fouling and increasing permeate flux, due to the stabilizing effect of mTG crosslinking on overall integrity of casein micelles, by forming highly stable iso-peptide covalent bonds within, and on the surface, resulting in a more compact micellar structure with restricted mobility of β-casein.

## Figures and Tables

**Table 1:** Composition, physicochemical and colour attributes of mTG crosslinked micellar casein concentrate feed used in microfiltration.

	CTR	TA	TB
Total solids (g/kg)	3.40 ^a^ ± 0.09	4.08 ^b^ ± 0.05	3.99 ^b^ ± 0.05
Total nitrogen (g/kg)	2.98 ^b^ ± 0.03	2.93 ^ab^ ± 0.05	2.87 ^a^ ± 0.06
Ash (g/kg)	0.28 ^a^ ± 0.02	0.28 ^a^ ± 0.05	0.29 ^a^ ± 0.07
pH	6.96 ^a^ ± 0.06	7.10 ^b^ ± 0.05	7.10 ^b^ ± 0.06
Viscosity (mPa·s)	3.15 ^a^ ± 0.35	3.44 ^a^ ± 0.78	3.13 ^a^ ± 0.22
L* value	81.3 ^b^ ± 1.01	80.3 ^a^ ± 0.92	82.2 ^b^ ± 0.54
a* value	−1.44 ^c^ ± 0.29	−2.08 ^a^ ± 0.14	−1.75 ^b^ ± 0.20
b* value	−0.62 ^c^ ± 0.05	−2.47 ^a^ ± 0.47	−1.38 ^b^ ± 0.17

Values are the means ± standard deviations of duplicate analysis from triplicate runs at each treatment. Different superscripted lower-case letters within a row indicate significant differences (*p* < 0.05). CTR: no enzyme treatment; TA: 5 °C/24 h; TB: 40 °C/90 min.

**Table 2:** Composition and physicochemical attributes of permeates generated from microfiltration of mTG crosslinked micellar casein concentrate feed at 5 ± 0.5 °C.

	CTR	TA	TB
Total solids (g/kg)	0.40 ^a^ ± 0.14	1.04 ^b^ ± 0.13	0.94 ^b^ ± 0.03
Total nitrogen (g/kg)	0.29 ^b^ ± 0.07	0.14 ^a^ ± 0.02	0.14 ^a^ ± 0.01
Ash (g/kg)	0.03 ^a^ ± 0.04	0.07 ^a^ ± 0.05	0.03 ^a^ ± 0.01
pH	7.00 ^a^ ± 0.07	7.13 ^b^ ± 0.05	7.13 ^b^ ± 0.10
Viscosity (mPa.s)	2.40 ^b^ ± 0.35	1.77 ^a^ ± 0.31	2.08 ^ab^ ± 0.27

Values are the means ± standard deviations of duplicate analysis from triplicate runs at each treatment. Different superscripted lowercase letters within a row indicate significant differences (*p* < 0.05). CTR: no enzyme treatment; TA: 5 °C/24 h; TB: 40 °C/90 min.

## Data Availability

The data presented in this study are available on request from the corresponding author.
